# Effects of Concentrate and Bypass Fat Supplementations on Growth Performance, Blood Profile, and Rearing Cost of Feedlot Buffaloes

**DOI:** 10.3390/ani11072105

**Published:** 2021-07-15

**Authors:** Amirul Faiz Mohd Azmi, Hafandi Ahmad, Norhariani Mohd Nor, Goh Yong Meng, Mohd Zamri Saad, Md Zuki Abu Bakar, Punimin Abdullah, Anuraga Jayanegara, Hasliza Abu Hassim

**Affiliations:** 1 Department of Veterinary Preclinical Sciences, Faculty of Veterinary Medicine, Universiti Putra Malaysia, UPM, Serdang 43400, Malaysia; amirulfaizazmi@gmail.com (A.F.M.A.); hafandi@upm.edu.my (H.A.); norhariani@upm.edu.my (N.M.N.); ymgoh@upm.edu.my (G.Y.M.); zuki@upm.edu.my (M.Z.A.B.); 2 Department of Veterinary Laboratory Diagnosis, Faculty of Veterinary Medicine, Universiti Putra Malaysia, UPM, Serdang 43400, Malaysia; mzamri@upm.edu.my; 3 Faculty of Science and Natural Resources, Universiti Malaysia Sabah, Jalan UMS, Kota Kinabalu 88400, Malaysia; puniminabdullah@ums.edu.my; 4 Animal Feed and Nutrition Modelling (AFENUE) Research Group, Department of Nutrition and Feed Technology, Faculty of Animal Science, IPB University, Bogor 16680, Indonesia; anuraga.jayanegara@gmail.com; 5 Laboratory of Sustainable Animal Production and Biodiversity, Institute of Tropical Agriculture and Food Security, Universiti Putra Malaysia, UPM, Serdang 43400, Malaysia

**Keywords:** blood biochemical, buffalo, cost analysis, growth performance, supplementation

## Abstract

**Simple Summary:**

Studies have shown that providing concentrate and bypass fat as feed supplements resulted in better performance of large ruminants. However, there is limited information about the effects of these supplements on the performance of buffaloes. This study evaluates the effects of concentrate and bypass fat supplementations on the growth performance, blood metabolites, and feeding cost of Murrah cross and Swamp buffaloes. Following diet supplementation, the feed intake, body weight, and body condition score were significantly improved without any side effects on the blood metabolites of both buffalo breeds. Although the mixture of concentrate and bypass fat supplement (26:4) used in this study was found to increase the cost of feed, overall, it resulted in a greater return.

**Abstract:**

This study investigates the effects of supplementation of the basal diet with concentrate and rumen bypass fat on the dry matter intake (DMI), growth performance, blood metabolites and hormonal changes, and the feeding cost of feedlot water buffaloes. Thirty-six healthy, three- to four-month-old male Murrah crossbred (n = 18) and Swamp (n = 18) buffaloes with a similar average initial body weight of 98.64 ± 1.93 kg were each randomly allocated into three dietary experimental groups. Buffaloes were fed with Diet A, which consisted of 100% *Brachiaria decumbens*, Diet B, consisting of 70% *Brachiaria decumbens* and 30% concentrate, and Diet C, consisting of 70% *Brachiaria decumbens*, 26% concentrate, and 4% rumen bypass fat for a period of 730 days. Feed intake was measured daily, while blood samples were collected for every eight months. Furthermore, body scores were noted prior to and at the end of the experimental period. The results showed that the average daily gain for buffaloes fed with Diet C was the highest. The DMI, BCS, FI, and FCR for the three groups showed significant (*p <* 0.05) differences, in the following order: Diet C > Diet B > Diet A. At the end of the two-year feeding trial, buffaloes fed with Diet B had significantly (*p <* 0.05) higher cholesterol levels than Diet A and Diet C. In addition, buffaloes fed with Diet C had significantly (*p <* 0.05) higher levels of serum total protein, growth hormone, and insulin-like growth factor-I hormone compared to Diet A and Diet B. On the other hand, buffaloes fed with Diet B and Diet C showed significant (*p <* 0.05) decrease in glucose levels. Supplemented diet improved the buffalos’ weight gain to achieve the market weight in a shorter period of time, thus, giving farmers a greater return. In conclusion, concentrate and bypass fat supplementations in the diet of water buffaloes improved the growth performance without adverse effect on the blood metabolites, which enabled better farmer profitability.

## 1. Introduction

Energy and protein are important constituents of animal diets. They play vital roles in the production and reproduction of animals. Therefore, nutrient requirements that are recommended by the National Research Council [1] are widely referred to when formulating diets for ruminants. However, the nutrient requirement equations presented by the NRC are mostly based on the requirements of cattle (*Bos taurus*). In general, the nutrient requirements of buffaloes are different from cattle, mainly due to the differences in climatic adaptability, nutrient utilization [2] and the digestibility of each nutrient in the feed [3]. Therefore, it is challenging to formulate diets for buffaloes due to limited references on the nutrient requirements and diet formulation for buffaloes (*Bubalus bubalis*).

Buffaloes are important to beef and dairy animals in several parts of the world, particularly in the tropical and subtropical regions of the globe. Cost-effective productions of quality buffalo beef and milk depend on accurate information on the buffaloes’ energy and protein requirements. In many developing countries, buffaloes are largely fed on natural pastures for survival, consisting of poor-quality roughages with low energy and high fibre [4]. This eventually resulted in poor growth [5], delayed age at puberty, low calving rate, and poor reproductive performance [6]. Nevertheless, the slow growth rate could be enhanced cost-effectively by proper feed and feeding, which include diet supplementation. Indeed, previous studies have reported that improving feed formulation by including concentrate supplementation for Murrah cross and Swamp buffaloes resulted in better growth and reproductive performances [7,8] that eventually resulted in greater returns to the farmer [8].

In livestock farming, the cost of feeding accounted for between 63% and 84% of the total cost of production and determined the economic viability of the livestock production system [9]. Furthermore, the late age-at-puberty and the delay in reaching market weight due to improper feeding increased farm operation costs [10]. Therefore, feeding buffaloes according to their protein and energy requirements is key to enhancing profitability [11]. The use of supplements in diet either as an individual concentrate or as part of a balanced concentrate mixture with bypass fat supplement is a widely observed practice, particularly in cattle farming. It has been shown that supplementation of concentrate and bypass fat with fresh grasses significantly improves feed intake and livestock performance [12,13]. The supplementation increases energy, proteins, minerals, and vitamins intakes, and with good quality forage, helps to overcome the problem of low palatability [10], leading to better production [14,15].

Nevertheless, long-term feeding of high concentrate diet decreases rumen pH [16], leading to a chronic disorder known as subacute ruminal acidosis [17]. However, the growth potential of calves could be fully exploited by incorporating bypass fat supplements in the ration. According to previous studies [18], the recommended inclusion of fat is between 2 and 3% for lactating animals and 10 and 15% of dry matter intake (DMI) (800 to 1000 g/day) for growing animals without any adverse effect on nutrient utilization [16,19]. Indeed, determining the appropriate level of supplementation is one of the important factors that ensures the growth and health of buffaloes. The improper ratio of supplementation might be detrimental to the health and productivity of the animals due to the increase in levels of cholesterol and triglyceride and eventually might create adverse effects on the health of human consumers [19]. Furthermore, scientific reports on the effect of feeding concentrate and bypass fat, especially on blood profile and feed cost analysis, in Murrah cross and Swamp buffaloes are scanty. Therefore, this study was conducted to assess the effects of dietary supplementation of concentrate and bypass fat on growth performance, serum biochemical, and hormonal profiles in Murrah cross and Swamp buffaloes, as well as on the cost of feeding.

## 2. Materials and Methods

### 2.1. Statement of Animals Rights

The study was performed and managed according to the Animal Utilization Protocol (AUP), Institutional Animal Care and Use Committee (IACUC), Universiti Putra Malaysia (Approval No. UPM/IACUC/AUP-017/2018, on 8 January 2018). Samplings from the experimental animals were strictly conducted under veterinary supervision.

### 2.2. Study Area

This study was conducted at the Buffalo Breeding and Research Centre, Sabah, Malaysia (Coordinate 5°30′ N, 117°7′ E). The farm consisted of 749 acres of land with a total of 405 heads of buffaloes. Two types of buffalo breeds were available on this farm, the Swamp and the Murrah × Swamp crossbred, representing 54% and 46% of the total buffalo population, respectively. The crossbred animals were the products of breeding pure Murrah males with Swamp females.

The 398.5 acres of pastureland were planted with establishing pasture, the *Brachiaria decumbens.* The Swamp and Murrah crossbred buffaloes in this farm were kept separated in different paddocks. Wallowing areas were available in each paddock, and drinking water was available ad libitum. The buffaloes were kept extensively and were free to graze all day within the paddocks that were enclosed by barbed wire. The farm practiced an extensive one-month rotational grazing system without feed supplementation, and the intervals were determined according to the size of each paddock to prevent over-grazing. This farm practiced natural breeding with a male to female ratio of 1:20. Breeding season was between November and January each year, and pregnancy diagnosis was carried out every three months following breeding.

### 2.3. Experimental Animals

A total of thirty-six male buffaloes consisting of Swamp (n = 18) and Murrah cross (n = 18) buffaloes of approximately 3 months old and with an average body weight of 98.64 ± 1.93 kg were randomly divided into three treatment groups with 6 animals per group. Prior to the onset of the experiment, a proper physical examination was conducted for each buffalo. Then, all buffaloes were weighed and treated against ecto- and endoparasites.

### 2.4. Experimental Design

The study was a completely randomized 2 × 3 factorial arrangement with three treatment diets, two breeds, and six replicates per treatment. Daily feed supply was calculated at 3% body weight (based on dry matter of total mixed ration), given in two equal portions at 07:00 h and 17:00 h [20]. The buffaloes were allowed a 14-day adjustment period to the respective diet before the start of the experiment. Three total mixed rations (TMR) were prepared that contained three components, namely, *Brachiaria decumbens* grass (G), commercial concentrate (C) that composed of corn grain (25.0%), palm kernel cake (32.0%), rice bran (18.0%), soya bean meal (19.7%), calcium carbonate (1.0%), molasses (2.8%), vitamin-mineral premix (0.3%), sodium chloride (0.6%), and dicalcium phosphate (0.6%), and bypass fat (B), which was the OPTI-FAT F8016RXP-rumen bypass supplement from fractionated palm fat without trans-fat. The nutritional composition of grass, concentrate, and bypass fat are presented in Table 1.

At the start of the experiment, buffaloes of group Diet A (control) were fed with 100% *Brachiaria decumbens* grass without supplementation. For Diet B, buffaloes were fed 70% grass with 30% concentrate, while for Diet C they were fed 70% grass, 26% concentrate, and 4% bypass fat as summarized in Table 2 [1,21,22]. Diets were supplied twice daily in the form of a total mix ration in which the grasses were offered as cut and carry and the concentrate and bypass fat were mixed according to the ration. The buffaloes were housed in individual pens with free access to clear drinking water and mineral blocks. The feeding trial lasted for 2 years. The area was 30 m^2^ per animal, mostly with a compacted dirt floor, while the area close to the feeder was covered with concrete. The feeders were vinyl type and were placed transversely on the upper part of the pens, while the drinkers were located at the divider between two pens.

The total mixed ration that consists of grass, concentrate, and bypass fat was analysed for nutritional composition according to the method of the Association of Official Analytical Chemists (AOAC) [23,24] and the results are summarized in Table 1 and Table 2.

### 2.5. Data Collection

Diet leftovers were weighed daily prior to the morning feeding to determine the average dry matter intake of each treatment. The intake was the difference between the amount of feed offered and the feed refused. The body weight was recorded before the start of the experiment and at three-monthly intervals, prior to morning feeding. The average daily gain was determined by dividing the increase in body weight over the experimental period by the length of the experimental period. The feed conversion ratio (FCR) was determined by measuring the amount of feed intake (kg DM) per kg of body weight gain during the experimental period. The body condition score of each buffalo was determined using a scale of 1 to 5 and was scored at the start and at the end of the experiment by evaluating the eight locations of the animal’s body as described by Roche et al. and Anitha et al. [25,26].

### 2.6. Blood and Serum Collection

Blood samples were collected from each buffalo prior to the start of feeding trial and every eight months until end of the feeding trial, approximately 1 h before the morning feeding. A total of 5 mL blood samples were collected in plain and EDTA Vacutainer tubes. The samples were cooled on ice and centrifuged within 24 h at 1000× *g* for 20 min. The serum and plasma were stored at −20 °C until analysis. The serum samples were used for serum biochemical analysis, including glucose, cholesterol, total protein, urea, and triglyceride, using a blood chemistry analyser (Siemens Dimension Xpand Plus, USA). Blood samples in EDTA tubes were used to determine the levels of growth hormone (GH) and insulin-like growth factor-I (IGF-I) using ELISA kits (Cloud-Clone Corporation, Wuhan, China) according to the manufacturer’s recommendation and run using the microwell method. The kit had a sensitivity and inter- and intra-run precision coefficient of variations for IGF-I of 1.56 ng/mL, <12%, and <10%, respectively, and GH of 0.312 ng/mL, <12%, and <10%, respectively. All plates were read using a computerized automated microplate ELISA reader (Infinite 200 series, TECAN). All measurements were made in one run with triplicate for each sample.

### 2.7. Cost of Feeding

The economic aspect of feedlot buffalo rearing was calculated based on feeding cost/kg live weight gain [27,28] at an exchange rate of 1 USD = 4.07 MYR. To calculate the total operational cost, it was assumed that the cost of feeding represented 87% of the total cost of the activity [29], and the cost of feeding comprised of the costs of basal diet and supplementations (concentrate and bypass fat) [30]. At the time of the study, the values of the feedstuffs (MYR/kg) were 0.23 MYR (0.06 USD) for *Brachiaria decumbens*, 1.11 MYR (0.27 USD) for concentrate, and 3.82 MYR (0.94 USD) for bypass fat. The 2-year management cost of 158.50 MYR (38.94 USD) per animal was added, which included the 0.50 MYR (0.12 USD) cost of deworming, 2.00 MYR (0.49 USD) for an ID tag, and 156.00 MYR (38.33 USD) for fertilizer. The average price of live weight buffalo in Malaysia was 14.60 MYR/kg (3.59 USD). The income through daily live weight gain, the cost of feed per day, and the net income through live weight in the 2-year study was calculated as below [27,28,30]:

Income live weight gain = Average daily gain × Current price of live weight (RM 14.60/kg)

Cost of feed per day = Current price of feed × Dry matter intake (based on 3% of body weight)

Net profit live weight gain = Income live weight gain in 2 years − Cost of feeding in 2 years − Management cost per animal in 2 years

Net profit live weight = Income live weight in 2 years − Cost of feeding in 2 years − Management cost per animal in 2 years

### 2.8. Statistical Analysis

All data were collected and recorded using Microsoft Excel and analyzed using the software package SPSS (Statistical Package for the Social Science 25.0, Inc., Chicago, IL, USA). Comparisons between breed, diet, and the interactions between breed and diet were performed using general linear model (GLM) procedures according to a 2 × 3 factorial arrangement in a completely randomized design (CRD). The resulting *p*-values were corrected using Tukey’s test to identify significant differences between the treatments. Linear mixed effects models were utilized with both ‘Diets’ and ‘Breed’ as fixed effects to capture the appropriate structure for GLM, while the feed intake, body weight pattern and gain, FCR, ADG, body condition score, serum biochemical, hormonal profiles, and cost of feeding were considered as a random effect. Furthermore, all data were also analysed using a GLM model for repeated measure procedure with treatment as the between subjects’ main effect and period of sampling (months) as the within subject factor. For all the statistical tests used, results were considered significant at *p* ≤ 0.05; differences between means were tested using the least significant difference. All procedures were carried out as per Snedecor and Cochran [31].

## 3. Results

### 3.1. Dry Matter Intake

**Figure 1:**
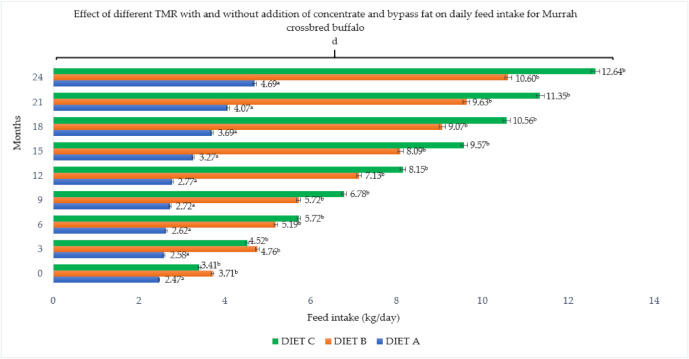
Effect of different TMR with and without addition of concentrate and bypass fat on daily feed intake for Murrah cross buffalo. Diet A (control)—(100% *Brachiaria decumbens)*; Diet B—(70% *Brachiaria decumbens* + 30% concentrate); Diet C—(70% *Brachiaria decumbens* + 26% concentrate + 4% bypass fat); the values are presented as mean; ^a,b^ different superscripts indicate significant difference on daily feed intake for Murrah crossbred buffalo at *p <* 0.05; ^d^ indicating significant (*p <* 0.05) difference comparing between months and feed intake.

**Figure 2:**
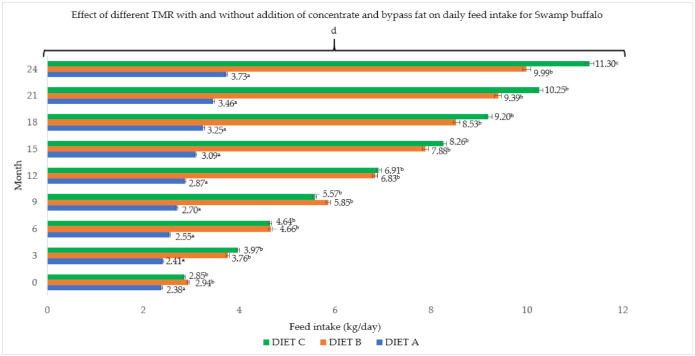
Effect of different TMR with and without addition of concentrate and bypass fat on daily feed intake for Swamp buffalo. Diet A (control)—(100% *Brachiaria decumbens)*; Diet B—(70% *Brachiaria decumbens* + 30% concentrate); Diet C—(70% *Brachiaria decumbens* + 26% concentrate + 4% bypass fat); the values are presented as mean; ^a,b,c^ different superscripts indicate significant difference on daily feed intake for Swamp buffalo at *p <* 0.05; ^d^ indicating significant (*p <* 0.05) difference comparing between months and feed intake.

The concentrate and bypass fat supplementations in the basal diet resulted in a significant (*p <* 0.05) effect and gradual changes in both buffalo breeds (Figure 1 and Figure 2). Total dry matter intake per day ranged between 4.70 and 12.64 kg/day for Murrah cross and between 3.72 and 11.30 kg/day for Swamp buffaloes, which was significantly (*p <* 0.05) influenced by the dietary treatment and level of supplement. Similarly, higher daily intake was observed in Murrah cross that were fed with Diet B and C. Both breeds fed with supplemented diets showed between 16.32 and 19.58 kcal ME/kg higher dry matter intake than the control group (Diet A) with 15.89 kcal ME/kg. There was no significant (*p* > 0.05) difference in the dry matter intake between the breeds, but a significant (*p <* 0.05) correlation was observed between months and dry matter intake for each breed during this two-year feeding trial.

### 3.2. Body Weight Pattern

**Figure 3:**
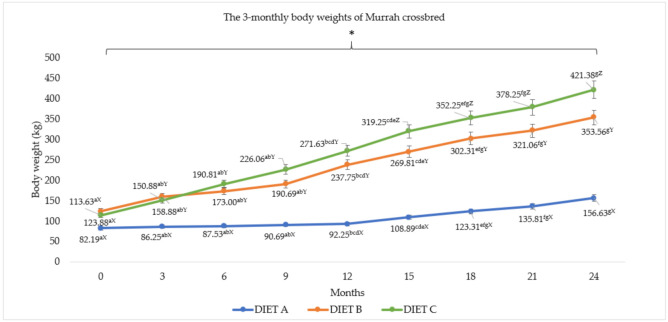
The three-monthly body weight (Mean ± SEM) of Murrah cross. Diet A (control)—(100% *Brachiaria decumbens)*; Diet B—(70% *Brachiaria decumbens* + 30% concentrate); Diet C—(70% *Brachiaria decumbens* + 26% concentrate + 4% bypass fat); the values are presented as mean; ^a,b,c,d,e,f,g^ different superscripts indicate significant difference of three-monthly body weight of Murrah crossbred buffalo for each Diet A, B, and C at *p <* 0.05; ^X,Y,Z^ different superscripts indicate significant difference between Diet A, B, and C for each three month interval of feeding at *p <* 0.05; * indicating significant (*p <* 0.05) difference comparing body weight and month.

**Figure 4:**
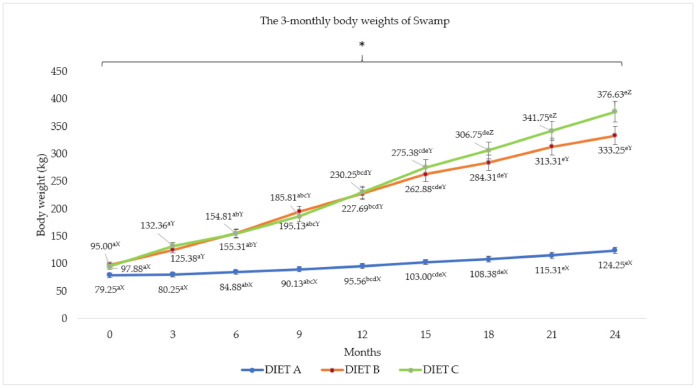
The three-monthly body weight (Mean ± SEM) of Swamp. Diet A (control)—(100% *Brachiaria decumbens)*; Diet B—(70% *Brachiaria decumbens* + 30% concentrate); Diet C—(70% *Brachiaria decumbens* + 26% concentrate + 4% bypass fat); the values are presented as mean; ^a,b,c,d,e^ different superscripts indicate significant difference of three-monthly bodyweight of Swamp buffalo for each Diet A, B, and C at *p <* 0.05; ^X,Y,Z^ different superscripts indicate significant difference between Diet A, B, and C for each three month interval of feeding at *p <* 0.05; * indicating significant (*p <* 0.05) difference comparing body weight and month.

The bodyweight patterns for Murrah cross and Swamp buffaloes are presented in Figure 3 and Figure 4. The effects of different diet on body weight of both buffalo breeds were highly significant (*p <* 0.05) throughout the experimental period. Higher body weight patterns were observed in both Murrah cross and Swamp buffaloes fed with Diet C containing 26% concentrate and 4% bypass fat, followed by Diet B and A. In general, the crossbreed showed significantly (*p <* 0.05) higher body weights than the Swamp buffaloes. The targeted 250 kg market weight was achieved in 12 months for Murrah cross and in 15 months for Swamp buffaloes that were fed with diet containing supplementations. In addition, there was a significant (*p <* 0.05) correlation between diet and month for the body weight pattern.

### 3.3. Average Daily Gain (ADG)

**Figure 5:**
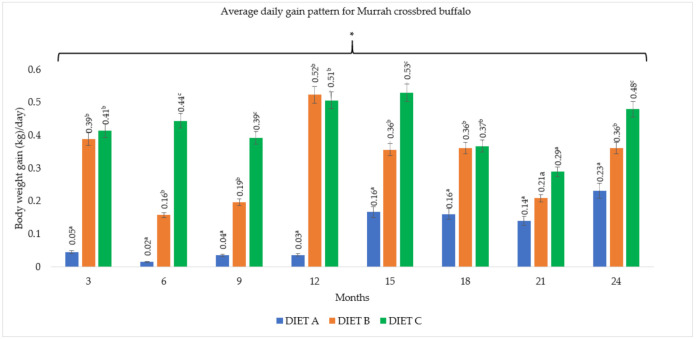
The average daily gain pattern at three-month intervals for Murrah crossbred buffaloes. Diet A (control)—(100% *Brachiaria decumbens)*; Diet B—(70% *Brachiaria decumbens* + 30% concentrate); Diet C—(70% *Brachiaria decumbens* + 26% concentrate + 4% bypass fat); the values are presented as mean; ^a,b,c^ different superscripts indicate significant difference of average daily gain pattern for Murrah crossbred buffalo at *p <* 0.05; * indicating significant (*p <* 0.05) difference comparing body weight gain and month.

**Figure 6:**
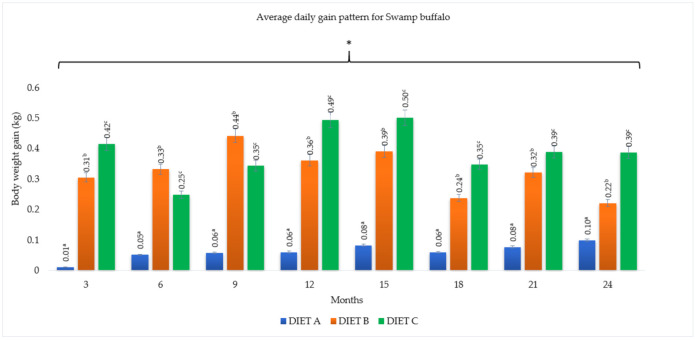
Effect of different diet on average daily gain at three-month intervals for Swamp buffalo. Diet A (control)—(100% *Brachiaria decumbens)*; Diet B—(70% *Brachiaria decumbens* + 30% concentrate); Diet C—(70% *Brachiaria decumbens* + 26% concentrate + 4% bypass fat); the values are presented as mean; ^a,b,c^ different superscripts indicate significant difference of average daily gain pattern for Swamp buffalo at *p <* 0.05; * indicating significant (*p <* 0.05) difference comparing body weight gain and month.

The average daily gain of the buffaloes fed with the three dietary treatments are summarized in Figure 5 and Figure 6. Buffaloes that were fed with Diet B and C showed significantly higher (*p <* 0.05) ADG in each of the three monthly intervals than buffaloes fed with Diet A (control). The mean daily gain for crossbred buffaloes fed with Diet A, B, and C throughout the two-year study were 0.10, 0.32, and 0.42 kg/day, respectively. On the other hand, the mean ADG for Swamp buffaloes were 0.06, 0.32, 0.39 kg/day, respectively. There was no correlation (*p >* 0.05) between the diet and breed for average daily weight gain.

The highest average daily weight gain was observed during the first 12-month period of the study, and the lowest was between 18 and 21 months of the study. The ADG range of buffaloes fed with supplement between 12 and 15 months was 0.51 kg/day to 0.53 kg/day for Murrah cross and 0.49 kg/day to 0.50 kg/day for Swamp buffaloes (*p <* 0.05), respectively. Furthermore, between 18 and 21 months, the ADG was significantly (*p <* 0.05) lower with 0.21 kg/day to 0.37 kg/day for Murrah cross and 0.24 kg/day to 0.39 kg/day for Swamp buffaloes, respectively. However, there was no correlation (*p >* 0.05) between body weight gain and month of the year.

### 3.4. Overall Intake and Growth Performance

The body weight gain (BWG), average daily gain (ADG), and feed conversion ratio (FCR) were significantly (*p <* 0.05) different between the different dietary treatment groups (Table 3). However, only the final body weight recorded a significant (*p <* 0.05) difference between the breeds. Average feed intake (kg/day) for Murrah cross and Swamp buffaloes fed with Diet A, B, and C during the two-year trial was 2.99 vs. 2.80, 6.57 vs. 6.15, and 7.41 vs. 6.37, respectively, which were statistically (*p <* 0.05) significant for diet but not breed. Murrah cross significantly (*p <* 0.05) had a 10.46% increase in final body weight when compared to Swamp breed. Furthermore, total BWG and ADG of buffaloes fed with supplement were significantly (*p <* 0.05) higher by three- to four-folds for Murrah cross and five- to six-folds for Swamp buffaloes as compared to the control diet. In general, the total feed intake, daily feed intake, total BWG, and ADG for Murrah cross were higher than Swamp buffaloes, and supplemented diets (Diet C and B) resulted in better growth performance.

The higher BWG among Murrah cross and Swamp buffaloes resulted in different FCR for the different diets (*p <* 0.05). The feed conversion ratio for buffaloes was reduced by between 31.08% and 42.26% for Murrah cross and between 63.32% and 68.16% for Swamp buffaloes when fed Diet B and C. The buffaloes were able to reach the targeted market weight of 250 kg between 9 and 12 months for Murrah cross and between 12 and 15 months for Swamp buffaloes when fed Diet C, but took between 12 and 15 months for both Murrah cross and Swamp buffaloes fed with Diet B. Moreover, the buffaloes were able to reach the targeted breeding weight of 350 kg in between 15 and 18 months for Murrah cross and between 21 and 24 months for Swamp buffaloes fed with Diet C and between 21 and 24 months and 24 to 26 months for Murrah cross and Swamp buffaloes fed with Diet B, respectively. However, Murrah cross and Swamp buffaloes fed with Diet A reached neither market weight nor breeding weight within the 24-month feeding trial. Moreover, there were correlations (*p <* 0.05) between diet and breed for total intake, initial body weight, FI, and FCR.

### 3.5. Body Condition Score

**Figure 7:**
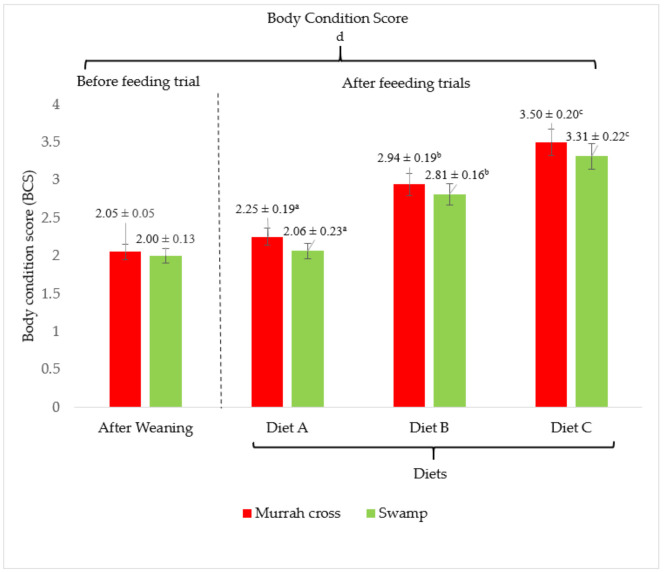
Effect of different diet on body condition score (BCS) at the end of the two-year feeding trial for Murrah cross and Swamp buffalo. Diet A (control)—(100% *Brachiaria decumbens)*; Diet B—(70% *Brachiaria decumbens* + 30% concentrate); Diet C—(70% *Brachiaria decumbens* + 26% concentrate + 4% bypass fat); the values are presented as mean; ^a,b,c^ different superscripts indicate significant difference of average daily gain pattern for Swamp buffalo at *p <* 0.05; ^d^ indicating significant (*p <* 0.05) difference comparing body condition score between before and after feeding trials.

**Figure 8:**
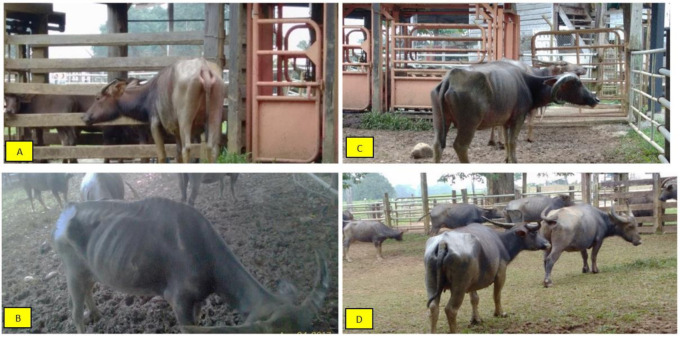
The body condition score of buffalo fed with and without supplemented diet. Notes: (**A**): after weaning calf (before feeding trials); (**B**): buffalo fed with Diet A; (**C**): buffalo fed with Diet B; (**D**): buffalo fed with Diet C.

The body condition score of the buffaloes fed a diet with and without supplementations are shown in Figure 7 and Figure 8. There were no significant (*p <* 0.05) differences in the BCS between buffalo breeds and no correlation (*p <* 0.05) between diet and breed. When fed Diet A, both breeds showed the lowest average BCS at 2.25 ± 0.19 and 2.06 ± 0.23 for crossbred and Swamp buffaloes, respectively. They showed visible backbone, hips, and shoulder, the ribs were faintly visible, and the tail head area was slightly recessed, significantly (*p <* 0.05) different from buffaloes fed with Diet B and C. Furthermore, there was a significant (*p <* 0.05) correlation between weaning and diets. On the other hand, buffaloes fed with Diets B and C showed a gradual but significant (*p <* 0.05) increase in the BCS. At the end of the study, the BCS ranged between 2.94 ± 0.19 and 3.50 ± 0.20 for Murrah cross and 2.81 ± 0.16 and 3.31 ± 0.22 for Swamp buffaloes. Compared with Diet A, both breeds fed with Diet B showed improvement in BCS: 30.7% for Murrah cross and 36.4% for Swamp buffaloes. Buffaloes that were fed with Diet C showed 55.6% and 60.7% improvement in BCS for Murrah cross and Swamp buffaloes, respectively. Buffaloes fed with supplemented diets (Diet B and C) were considered moderately lean where the hip bones were visible faintly, ribs were not visible, tail head area was not recessed, and body outline appeared smooth.

### 3.6. Serum Biochemical and Hormonal Profiles

The concentration of plasma glucose, cholesterol, total protein, urea, triglyceride, growth hormone (GH), and insulin like growth factor-I (IGF-I) following a two-year study are tabulated in Table 4 and Table 5. The blood glucose, cholesterol, GH, and IGF-I were significantly (*p <* 0.05) influenced by the supplemented diet, while other blood metabolites were not. All blood parameters were also shown to be significantly influenced by the period (*p <* 0.05). The blood glucose level in buffaloes fed with Diet B and C showed a significant (*p <* 0.05) decrease of between 15% and 16% compared with Diet A (control). Significant (*p <* 0.05) increments of cholesterol and triglyceride were observed in both breeds fed with Diet B compared with Diet C and A. Similarly, buffaloes fed with Diet C did not show any significant (*p <* 0.05) impact on blood profiles.

The GH and IGF-I were significantly (*p <* 0.05) highest in both breeds that were fed Diet C, followed by Diet B and Diet A (Table 5). The increased IGF-I levels in buffaloes fed with Diet B and C were between 22.4% and 28.4% for Murrah cross and between 22.0% and 25.6% for Swamp buffaloes. Meanwhile, the GH level was recorded between 27.5% and 31.0% for Murrah cross and between 14.9% and 25.6% for Swamp buffaloes, higher than the control diet (Diet A). Furthermore, there were insignificant correlations between diet, breed, and period in these parameters (*p >* 0.05).

### 3.7. Analysis of the Cost of Feeding

Table 6 shows the total costs of feed for Murrah cross and Swamp buffaloes in the two-year feeding trial. They were found to be comparable. The higher average daily intake of diets with supplementation by Murrah cross resulted in higher feed costs of between 2.17 MYR (0.53 USD) and 2.98 MYR (0.73 USD) per animal/day, while Swamp buffalo was between 2.02 MYR (0.50 USD) and 2.56 MYR (0.63 USD) per animal/day. Buffaloes fed with Diet A showed significantly (*p <* 0.05) fewer costs of feeding, between 0.46 MYR (0.11 USD) and 0.43 MYR (0.10 USD) per animal/day.

The costs of diets with supplementation were roughly five- to seven-fold higher than the diet without supplementation, following higher consumption of supplemented feed. However, the net profit after two years of the feeding trial showed that buffaloes fed with supplemented diets provided significantly (*p <* 0.05) higher returns that ranged between 1,319.24 MYR (324.14 USD) and 1,421.94 MYR (349.37 USD) per animal for Diet B and between 1,708.84 MYR (419.86 USD) and 2,013.12 MYR (494.62 USD) per animal for Diet C compared with control Diet A that ranged between 154.80 MYR (38.03 USD) and 176.25 MYR (43.31 USD) per animal. In addition, Murrah cross showed significantly (*p <* 0.05) greater returns than the Swamp buffaloes.

## 4. Discussion

Genetic improvements of buffaloes include the choice of breed, crossbreeding, and selection within breeds [35]. These selections aim to improve outputs such as better body weight gain, reproductive performance, and carcass traits [35]. However, the overall aim of large ruminant breeding is to improve profitability [35]: the shorter the period taken for buffalo to achieve the market weight, the more the return gained by the farmers. In this study, the Murrah crossbred showed significantly heavier bodyweight than Swamp buffaloes, similar to buffaloes in Indonesia [36], Thailand [37], and the Philippines [38]. This is because breed influences the bodyweight of buffalo calves [38]. Similarly, proper nutritional management greatly influences the bodyweight of animals [39]. Livestock farmers in most developing tropical countries were forced to maximize the limited feed resources for their livestock, resulting in significant inefficiency in the ruminant metabolic processes [40]. This affects the average daily gain and feed conversion ratio [41], and eventually the body weight. Therefore, the formulation of diets based on energy and protein intake per unit of live weight gain might give a similar performance pattern for growing animals, especially for buffalo calves.

The average body weight of weaned buffalo calves in the present study was 98.64 kg, which was higher than the 86.5 kg reported earlier [42]. A similar study revealed that approximately nine months were needed for the calves to achieve bodyweight of more than 100 kg, irrespective of the breed of the buffaloes [38]. However, both Swamp and crossbreeds showed rapid pre-weaning growth, although the crossbreeds showed significantly better growth, leading to a better overall daily weight gain of 0.89 kg/day, compared to 0.65 kg/day reported by Vaz et al. [42]. To achieve slaughter or breeding weight in a short period requires proper diet formulation. Supplementation in basal diets significantly (*p <* 0.05) affected the growth performance parameters such as ADG, BWG, and BCS. Therefore, the final body weight of the buffaloes in this two-year feeding trial reached between 350 and 400 kg in 12 to 15 months, which is suitable for slaughter [43]. However, Murrah cross was reported to reach slaughter weight much faster than Swamp buffaloes even when fed with the same diet. Furthermore, male buffalo calves were able to reach 350 kg body weight with an initial weight of 200 kg in a short fattening period of about four months [44].

One of the reasons for the better growth performance with the supplemented diets was the feed intake, which was better in the treatment groups [19]. In fact, when grazing cattle were offered concentrate supplement, they showed 0.9 kg/d higher total dry matter intake [45], similar to buffaloes fed with Diet B in this study. However, the improved feed intake by buffaloes fed with Diet C in this study disagreed with other studies that concluded that supplementation of rumen bypass fat at levels higher than 2.5% could reduce the DMI [46,47] due to the release of peptides in the gut as a response to a higher fat diet composition [48]. Furthermore, the addition of long-chain fatty acid capsulated with Ca salts at a high level was capable of depressing animal daily intake [49]. Thus, the inclusion of 5% to 20% of protected fat in diets could significantly decrease the feed intake of dairy cows [50], although no adverse effect was observed on rumen fermentation [51]. Similarly, Duckett et al. [52] reported a decrease in feed intake even with 1% fat supplementation. Nevertheless, Fiorentini et al. [53] reported that higher intake of dry matter and organic matter were observed in animals fed with supplementation consisting of protected fat compared to animals fed with unprotected fat palm oil, linseed oil, and whole soybeans, as observed in this study. A previous study had shown that different percentages of bypass fat that ranged between 30% and 80% would not affect the rumen fermentation characteristics such as total volatile fatty acid, total nitrogen, ammonia, and apparent rumen degradability content [54]. Furthermore, Naik et al. [55] concluded that there was no difference in the apparent digestibility of dry matter, organic matter crude protein, total carbohydrate, NDF, ADF, cellulose except in ether extract, and hemicellulose in buffalo rumen. Indeed, the apparent digestibility of all nutrients except ether extract and hemicellulose did not give any changes at different levels of bypass fat due to non-interference of bypass fat with digestibility of nutrients due to its relatively stable nature and minimum dissociation in the rumen [56].

The pattern of ADG in this study was similar to previous studies that fed male buffalo calves with low, medium, and high energy diets that contained 90%, 100%, and 110% of NRC recommendations [57,58]. The ADG was recorded at 516, 559, and 607 g/day, respectively, and was influenced by the energy levels. However, a higher daily weight gain of 980 g/day for yearling buffalo calves compared to 420 g/day in our study has been reported and this might be due to the use of different buffalo breeds [59]. Furthermore, the variations in average daily gain of animals could also be affected by the differences in initial body weight, genetic resources, age, and nutritional management [60,61].

An important indicator of good weight gain and health status of livestock is the body condition score. The use of BCS to indicate adequate production management has the advantage of being fast, accurate, economical, and non-invasive [62]. In this study, body weight and BCS were found to have a positive correlation and be in accordance with the type of supplement added. Buffaloes that were offered concentrate and bypass supplements had higher BCS due to better body weight gain. Similarly, buffaloes fed with concentrate have been shown to have good BCS [63], while cattle fed bypass fat showed improved feed intake [64]. On the other hand, the addition of 4 kg/d to 8 kg/d of concentrates for five weeks was able to improve the body mass gain without changes in BCS of dairy cows [65], while another study revealed that additional bypass fat did not improve body weight gain and BCS [66]. The variation in results might be due to the feed, energy and protein sources, period of feeding trials, type of animals, breeds, and the age of animals [66].

In this study, blood profiles were within the normal range, no adverse effect was observed, and no clinical signs related to metabolic disorder were detected following diet supplementation in buffaloes. Feeding concentrate and bypass fat to growing buffalo calves had little impact on the blood total protein and cholesterol but lowered the level of blood glucose [28,67,68], which remained within the normal range of 1.97–5.13 mmol/L. The findings were similar to previous studies which reported a slight increase in total protein and lipid profiles in buffaloes supplemented with concentrate and bypass fat and contributed to positive energy balance [19] and suggested that nutrient supply could also influence the lipogenic enzyme activities [69,70]. The decreased blood glucose level was calculated to be between 43% and 52%, much higher than the previous report of 10.24% [71]. On the other hand, glucose levels negatively correlate with age, with a higher value at weaning age and a lower value at an older age. This is in relation with the increased intake of starter diet post-weaning [72], causing high ruminal fermentation that switched the energy reliance to volatile fatty acids leading to lower blood glucose level in advanced age [73,74]. Therefore, supplementation of fat in the diet for a short period of time (less than 100 days) resulted in a positive correlation with the increased blood glucose and cholesterol levels [19,75] following an enhanced uptake of dietary fatty acid [12]. According to Tyagi et al. [76], supplementing with concentrate and 2.5% of bypass fat did not give any change in blood cholesterol level in growing cows. However, in the present study, serum glucose and cholesterol levels showed a decreasing pattern after 24 months of the feeding trial. This may be due to animal studies facing a long-term period of feeding trial; thus, the animal was undergoing normal homeostasis as the pre-requisite for maintaining health [77].

Growth hormone and insulin-like growth factor-I are parts of the somato-trophic axis that have multifunctional roles in the metabolism and physiology of mammals [78]. A study showed that injecting young cattle with exogenous bovine GH increased the ADG levels; thus, ADG and BW had a positive correlation with serum GH and IGF-I [79]. However, there was no difference in the concentrations of GH between Murrah cross and Swamp buffaloes in this study; thus, breed had no significant effect on plasma GH levels [80,81], although a study found an association between GH concentration and breed of animals [82]. Nevertheless, the CP:ME ratio might influence the GH and IGF-I levels in Holstein heifers [32]. Therefore, a higher dietary energy level decreases the GH serum concentration [33]. Our study showed that GH and IGF-I worked well in promoting proteo-synthesis and lipolysis since both hormones were significantly high in Murrah cross and Swamp buffaloes compared to the control. We observed that the serum level of IGF-I increased with an increased dietary energy level, in agreement with previous studies in heifers and dairy cattle [34,83]. Furthermore, this study also showed that the experimental trial period significantly affected all blood and hormonal profiles [37].

In general, the crossbreeds had significantly heavier body weight than Swamp buffaloes from birth until 24 months old. A similar observation was made among buffaloes in Indonesia [36], Thailand [37], and the Philippines [43]. Body weights of buffalo calves are influenced by many factors such as feeding management [84], breeds of buffalo [85,86], and environmental factors [87]. Good feeding management improves farm husbandry and increases revenue for both Swamp and crossbred buffaloes [7,88]. In addition, crossbreeds are able to reach market weight much earlier than the Swamp buffaloes. However, early weaning is costlier for the farm due to the longer weaning-to-production period. This leads to a slightly higher additional cost of the crossbreeds. On the other hand, the birth weight could also affect the reproduction maturity of females [89], when the crossbreed tends to reach the age at first calving earlier than the Swamp buffaloes. Thus, the females can reproduce earlier and for a longer period, which brings more economic benefit to the farm. The earlier age of first calving also reduces the cost of rearing heifers. In addition, studies also showed that heterosis might have impacts on the growth and performance of buffaloes. It can come in three different forms, either individual, maternal, or paternal [90]. According to a recent study, individual heterosis is used in crossing between two breeds, which increased the performance of crossbred progeny relative to that of its purebred parents [91]. Furthermore, maternal and paternal heterosis increased the production of a cow above that of the average of her parent breeds, giving advantages in terms of improvement of reproduction, longevity, calf survivability, increased calf birth weight, shorter period of birth age puberty, and improved bull fertility [92]. However, the impact of the heterosis relies on the level of genetic differences between the original breed, whereby further variation between the two basic breeds causes the heterosis impact to be even greater [91]. Crossbreeding of pure Murrah and Swamp breeds is a common practice developed by farmers in Malaysia and other Asian countries, so that the crossbreeding can inherit superior traits possessed by their parents. The finding of this study is in agreement with previous studies that showed that crossbred buffalo have a greater performance of growth compared to purebred [7,8]. According to Shaari et al. [93], the crossbred Murrah in Malaysia was the product of crossbreeding between male Murrah and female Swamp buffaloes (exhibit 2n = 50 and 2n = 48 number of chromosomes, respectively), producing 49 chromosomes and has shared conserved regions of the genes from their parents. Indeed, a previous study also reported that analysis of mtDNA and phylogenetic trees showed Swamp buffaloes were genetically conserved and the crossbreeds were dominantly Swamp according to the maternal lineage using d-loop mtDNA [94]. Nevertheless, the crossbreed’s performance was better than Swamp buffaloes, especially regarding growth, meat, and milk production [8,94].

Nutrition is one of the production factors that reflects the total production and profit of a farm [95]. In this study, total feed cost was significantly (*p <* 0.05) increased for the diets with supplementation but resulted in a significant increase in BW; thus, the targeted selling price was significantly (*p <* 0.05) higher, as earlier observed [30]. The high cost was due to the additional cost of supplements added into the basal diet, where Diet C was three folds and Diet B showed two folds higher than the non-supplemented Diet A (control). Nevertheless, the two-year fattening resulted in significantly higher net profit for Diet C, followed by Diet B. Similar results were observed by Naik et al. [96] and Raval et al. [30].

## 5. Conclusions

Supplementation with concentrate and bypass fat produced positive effects on the performance of feedlot buffaloes of both breeds. In addition, the supplementations have no adverse effect on serum biochemistry and increased the hormones related to growth. Even though supplementation increased the feed cost per day, subsequently animals could reach the standard market and breeding weight faster and at a significantly younger age, resulting in better income for the farm. Subsequently, Murrah crossbred buffaloes showed a significantly better body condition score and body weight gain and thus reached market and breeding weight at a significantly younger age than Swamp buffaloes. Further studies should address the effect of concentrate and bypass fat supplementations on rumen fermentation, rumen microbial population, and the meat quality of buffaloes.

## Figures and Tables

**Table 1:** Nutritional composition of feedstuff (% DM basis).

Nutrient Composition	Diet Components		
	Grass (G)	Concentrate (C)	Bypass Fat (B)
DM ^1)^ (%)	90.34	90.36	99.75
Ash (% DM)	5.09	5.44	-
CF ^2)^ (% DM)	26.03	7.49	-
EE ^3)^ (% DM)	2.03	5.46	100
CP ^4)^ (% DM)	6.09	18.15	-
NDF ^5)^ (% DM)	64.27	56.87	-
ADF ^6)^ (% DM)	33.86	17.38	-
ADL ^7)^ (% DM)	3.55	2.96	-
NFC ^8)^ (% DM)	22.05	13.85	-
GE ^9)^ (MJ/kg)	11.79	15.74	37.65

Note: Data shown are the mean of triplicate analyses of each component. Three components of feedstuffs namely *Brachiaria decumbens* grass (G), commercial concentrate (C) (composition: corn grain (25.0%), palm kernel cake (32.0%), rice bran (18.0%), soya bean meal (19.7%), calcium carbonate (1.0%), molasses (2.8%), vitamin-mineral premix (0.3%), sodium chloride (0.6%), dicalcium phosphate (0.6%)), and bypass fat (B) (sources from calcium salt fractionated palm fat without trans-fat). Abbreviations: ^1)^ DM, dry matter; ^2)^ CF, crude fiber, ^3)^ EE, ether extract; ^4)^ CP, crude protein; ^5)^ NDF, neutral detergent fibre; ^6)^ ADF, acid detergent fibre; ^7)^ ADL, acid detergent lignin; ^8)^ NFC, non-fiber carbohydrate; ^9)^ GE, gross energy.

**Table 2:** Nutritional composition of experimental diets, based on a dry matter basis.

Ingredient	% Composition in TMR				
	Diet A	Diet B	Diet C		
*Brachiaria decumbens* (G)	100	70	70		
Concentrate (C)	-	30	26		
Bypass fat (B)	-	-	4		
**Nutrient**	**Estimated Content**			**SEM ^1)^**	***p*-Value**
DM ^2)^ (%)	90.34	90.31	91.60	0.24	0.374
Ash (% DM)	5.09	5.69	5.93	0.33	0.916
CF ^3)^ (% DM)	26.03 ^a^	23.73 ^b^	21.65 ^c^	0.66	<0.001
EE ^4)^ (% DM)	2.03 ^a^	2.92 ^b^	16.66 ^c^	2.87	<0.001
CP ^5)^ (% DM)	6.09 ^a^	8.08 ^b^	6.56 ^a^	0.36	0.012
NDF ^6)^ (% DM)	64.27 ^a^	57.96 ^b^	49.63 ^c^	2.17	<0.001
ADF ^7)^ (% DM)	33.86 ^a^	28.7 ^b^	26.65 ^c^	1.10	<0.001
ADL ^8)^ (% DM)	3.55	3.32	2.96	0.18	0.442
NFC ^9)^ (% DM)	22.05	24.84	20.81	2.29	0.074
GE (MJ/kg) ^10)^	11.07 ^a^	12.1 ^a^	14.59 ^b^	0.56	<0.001
Hemicellulose (% DM)	30.41 ^a^	29.25 ^a^	22.98 ^b^	1.24	0.002
Cellulose (% DM)	30.32 ^a^	25.38 ^b^	23.69 ^b^	1.04	0.001

Note: Diet A (control): 100% *Brachiaria decumbens*; Diet B: 70 *Brachiaria decumbens* + 30% concentrate; Diet C: 70% *Brachiaria decumbens* + 26% concentrate + 4% bypass fat. Data are the mean of triplicate analyses of each diet. ^a,b,c^ Means in the same row with different superscript are significantly different (*p <* 0.05). Abbreviations: ^1)^ SEM, standard error of the mean; ^2)^ DM, dry matter; ^3)^ CF, crude fiber; ^4)^ EE, ether extract; ^5)^ CP, crude protein; ^6)^ NDF, neutral detergent fiber; ^7)^ ADF, acid detergent fiber; ^8)^ ADL, acid detergent lignin; ^9)^ NFC, non-fibrous carbohydrate; ^10)^ GE, gross energy.

**Table 3:** Feed intake, body weight gain (BWG), average daily gain (ADG), and feed conversion ratio (FCR) of buffaloes fed with and without supplement over two-year experiment.

Attribute	Diet						Breed			*p*-Value		
	Murrah Cross			Swamp			Murrah Cross	Swamp				
Parameter	Diet A	Diet B	Diet C	Diet A	Diet B	Diet C			SEM	Diet	Breed	Interaction
Total intake (kg)	2183.12 ^a^	4798.91 ^b^	5407.43 ^c^	2043.23 ^a^	4487.06 ^b^	4649.74 ^b^	4129.82	3726.68	206.76	0.001	0.332	0.014
Initial BW (kg)	82.19	123.88	113.63	79.25	97.88	95.00	106.57	90.71	5.07	0.081	0.064	0.088
Final BW (kg)	156.63 ^a^	353.56 ^b^	421.38 ^c^	124.25 ^a^	333.25 ^b^	376.63 ^c^	310.52 ^y^	278.04 ^z^	34.54	<0.001	0.047	0.210
BWG (kg)	74.44 ^a^	229.69 ^b^	307.75 ^c^	45.00 ^a^	235.38 ^b^	281.63 ^c^	203.96	187.34	30.68	<0.001	0.582	0.054
ADG (kg/day)	0.10 ^a^	0.32 ^b^	0.42 ^c^	0.06 ^a^	0.32 ^b^	0.39 ^c^	0.28	0.26	0.04	<0.001	0.582	0.054
Feed intake (DM kg/day)												
*Brachiaria decumbens*	2.99	4.60	5.19	2.80	4.31	4.46	4.26	3.86	0.27	0.079	0.101	0.127
Concentrate	-	1.97	1.93	-	2.06	1.66	1.95	1.86	-	-	-	-
Bypass fat	-	-	0.29	-	-	0.25	0.29	0.25	-	-	-	-
Total feed intake per day (kg/day)	2.99 ^a^	6.57 ^b^	7.41 ^c^	2.80 ^a^	6.15 ^b^	6.37 ^b^	5.66	5.11	0.55	<0.001	0.309	0.014
FCR	30.57 ^a^	21.07 ^b^	17.65 ^b^	52.24 ^a^	19.16 ^b^	16.63 ^b^	23.10	29.34	3.87	<0.001	0.141	<0.001

Note: Diet A (control): 100% *Brachiaria decumbens*; Diet B: 70% *Brachiaria decumbens* + 30% concentrate); Diet C: 70% *Brachiaria decumbens* + 26% concentrate + 4% bypass fat; ^a,b,c,y,z^ means with different superscript letters in the same column are significantly different at *p <* 0.05. Abbreviations: SEM: standard error of means, BW: body weight, BWG: body weight gained, ADG: average daily gained, FI: feed intake, FCR: feed conversion ratio.

**Table 4:** Effect of different diet on serum biochemical and hormonal profiles at the end of the two-year feeding trial for both buffalo.

Attribute	Diet						Breed		SEM	Ref. Interval	Ref.
	Murrah Cross			Swamp			Murrah Cross	Swamp			
Diets	Diet A	Diet B	Diet C	Diet A	Diet B	Diet C					
Glucose (mmol/L)											
0 month	4.80	5.10	5.30	5.40	5.20	5.10	5.07	5.23	0.09	1.97–5.13	Abd Ellah et al. [32]
8 months	4.55 ^a^	4.00 ^b^	4.10 ^b^	5.00 ^a^	4.97 ^a^	4.86 ^b^	4.22	4.94	0.18		
16 months	4.29 ^a^	3.82 ^b^	3.85 ^b^	4.11 ^a^	4.14 ^a^	3.92 ^b^	3.99	4.39	0.08		
24 months	4.23 ^a^	3.53 ^b^	3.58 ^c^	5.43 ^a^	4.53 ^b^	3.58 ^c^	3.78	4.51	0.17		
Overall mean	4.47	4.11	4.21	4.76	4.47	4.40	4.26	4.54	0.09		
Cholesterol (mmol/L)											
0 month	3.87	3.95	3.89	3.52	2.99	3.14	3.90	3.22	0.17	0.75–2.67	
8 months	3.14 ^a^	2.39 ^b^	2.32 ^b^	2.90 ^a^	2.43 ^b^	2.55^b^	2.62	2.63	0.13		
16 months	2.56 ^a^	1.86 ^b^	1.79 ^b^	2.10 ^a^	2.44 ^b^	2.35^b^	2.07	2.30	0.13		
24 months	1.68 ^a^	2.00 ^b^	1.98 ^c^	2.38 ^a^	2.50 ^b^	2.13^c^	1.89	2.34	0.13		
Overall mean	2.81 ^a^	2.55 ^b^	2.45 ^b^	2.73 ^a^	2.59 ^b^	2.54^b^	2.60	2.62	0.06		
Total protein (g/L)											
0 month	85.23	83.15	86.76	78.37	81.64	80.93	85.05	80.31	1.24	56.30–98.30	
8 months	72.80	77.31	79.34	80.15	81.05	81.99	76.48	81.06	1.36		
16 months	75.46	78.95	83.03	81.64	80.57	85.34	79.15	82.52	1.40		
24 months	79.65^a^	79.53 ^a^	89.14 ^b^	79.70 ^a^	78.00 ^a^	89.08 ^b^	82.77	82.26	2.10		
Overall mean	78.29^a^	79.74 ^a^	84.57 ^b^	79.97 ^a^	80.32 ^a^	84.34 ^b^	80.87	81.54	1.07		
Urea (mmol/L)											
0 month	6.3	6.77	6.61	6.97	6.52	6.89	6.56	6.79	0.10	5.40–21.24	
8 months	6.01	6.62	6.50	6.33	6.44	6.48	6.38	6.42	0.09		
16 months	5.90	6.39	6.43	5.55	5.89	5.74	6.24	5.73	0.15		
24 months	5.80	5.98	6.38	5.05	5.20	5.38	6.05	5.21	0.21		
Overall mean	6.00	6.44	6.48	5.98	6.01	6.12	6.31	6.04	0.09		
Triglyceride (mmol/L)											
0 month	0.33	0.31	0.33	0.18	0.21	0.19	0.32	0.19	0.03	0.05–0.65	
8 months	0.27	0.24	0.24	0.16	0.24	0.19	0.25	0.20	0.02		
16 months	0.21	0.18	0.20	0.15	0.25	0.17	0.20	0.19	0.01		
24 months	0.17	0.13	0.18	0.20	0.28	0.14	0.16	0.21	0.02		
Overall mean	0.26	0.22	0.24	0.17	0.25	0.17	0.24	0.20	0.01		
IGF-I (ng/mL)											
0 month	114.01	116.74	112.49	108.89	107.08	108.44	114.41	108.14	1.53	117–300	Ashmawy [33]
8 months	116.43 ^a^	128.35 ^b^	133.09 ^b^	110.72 ^a^	124.34 ^b^	124.06 ^b^	125.96	119.71	3.30		
16 months	119.25 ^a^	147.52 ^b^	152.11 ^b^	116.49 ^a^	144.42 ^b^	149.38 ^b^	139.63	136.76	6.52		
24 months	122.80 ^a^	158.30 ^b^	171.61 ^c^	119.44 ^a^	153.93 ^b^	159.57 ^c^	150.90	144.31	8.72		
Overall mean	118.12 ^a^	137.73 ^b^	142.33 ^b^	113.89 ^a^	132.45 ^b^	135.36 ^b^	132.73	127.23	4.65		
GH (ng/mL)											
0 month	1.92	1.93	1.91	1.6	1.66	1.59	1.92	1.62	0.07	0.05–17.00	Mishra et al. [34]
8 months	1.91 ^a^	2.11 ^b^	2.30 ^b^	1.72 ^a^	1.89 ^b^	1.91 ^b^	2.11	1.84	0.08		
16 months	1.86 ^a^	2.37 ^b^	2.54 ^c^	1.78 ^a^	2.07 ^b^	2.14 ^c^	2.26	2.00	0.12		
24 months	1.87 ^a^	2.58 ^b^	2.71 ^c^	1.83 ^a^	2.15 ^b^	2.46 ^c^	2.39	2.15	0.15		
Overall mean	1.89 ^a^	2.25 ^b^	2.37 ^b^	1.73 ^a^	1.94 ^b^	2.03 ^c^	2.17	1.90	0.10		

Note: Diet A (control): 100% *Brachiaria decumbens*; Diet B: 70% *Brachiaria decumbens* + 30% concentrate); Diet C: 70% *Brachiaria decumbens* + 26% concentrate + 4% bypass fat; the values are presented as mean ± SEM (standard error of mean); ^a,b,c^ means with different superscript letters in the same column are significantly different at *p <* 0.05. Abbreviations: SEM: standard error of means, GH: growth hormone, IGF-I: insulin-like growth factor-I.

**Table 5:** The significant values for the effect of different diet on serum biochemical and hormonal profiles for both buffalo.

	*p*-Value			Interaction			
Parameters	Diet	Breed	Period	Diet * Breed	Breed * Period	Diet * Period	Diet * Breed * Period
Glucose (mmol/L)	<0.001	0.674	0.04	0.963	0.104	0.051	0.682
Cholesterol (mmol/L)	<0.001	0.266	<0.001	0.757	0.095	0.080	0.466
Total Protein (g/L)	0.049	0.469	<0.001	0.983	0.295	0.361	0.301
Urea (mmol/L)	0.341	0.299	<0.001	0.246	0.118	0.873	0.215
Triglyceride (mmol/L)	0.066	0.789	<0.001	0.246	0.316	0.078	0.961
Hormones							0.634
IGF-1 (ng/mL)	0.017	0.592	<0.001	0.752	0.303	0.077	0.462
GH (ng/mL)	<0.001	0.076	<0.001	0.665	0.081	0.056	0.075

Note: * indicates interaction between parameter studied.

**Table 6:** Cost analysis (RM) of buffaloes fed with different supplemented diet.

	Diet						Breed					
	Murrah Cross			Swamp			Murrah Cross	Swamp	SEM	*p*-Value		
	A	B	C	A	B	C				Diet	Breed	Interaction
A. Income from live weight gain (MYR/day/animal)	1.49 ^a^	4.59 ^b^	6.16 ^c^	0.90 ^a^	4.71 ^b^	5.63 ^c^	4.08 ^y^	3.75 ^z^	0.61	<0.001	0.012	0.001
B. Cost of feeding (RM/day)												
*Brachiaria* grass	0.46	0.71	0.80	0.43	0.66	0.68	0.66	0.59				
Concentrate	-	1.46	1.43	-	1.36	1.23	1.45	1.30				
Bypass fat	-	-	0.75	-	-	0.65	0.75	0.65				
Total cost of average daily DMI (MYR/day/animal)	0.46 ^a^	2.17 ^b^	2.98 ^c^	0.43 ^a^	2.02 ^b^	2.56 ^c^	1.87	1.67	0.30	<0.001	0.505	0.044
C. Fixed cost in 2 years												
Deworming	0.50	0.50	0.50	0.50	0.50	0.50	0.50	0.50				
ID tag	2.00	2.00	2.00	2.00	2.00	2.00	2.00	2.00				
Fertilizer	156.00	156.00	156.00	156.00	156.00	156.00	156.00	156.00				
Total (MYR/2year/animal)	158.50	158.50	158.50	158.50	158.50	158.50	158.50	158.50				
D. Gross return over feed cost (RM/day/animal) (A-B)	1.03 ^a^	2.43 ^b^	3.18 ^c^	0.47 ^a^	2.68 ^b^	3.08 ^c^	2.21	2.08	0.32	<0.001	0.289	0.042
E. Net profit from live weight gain for 2 years (MYR/animal)	176.25 ^a^	1421.94 ^b^	2013.12 ^c^	154.80 ^a^	1319.24 ^b^	1708.84 ^c^	1203.77 ^y^	1060.96 ^z^	220.33	<0.001	<0.001	<0.001
F. Net profit from live weight for 2 years (MYR/animal)	1793.48 ^a^	3423.07 ^b^	3821.95 ^c^	1342.26 ^a^	3229.21 ^b^	3472.89 ^c^	3012.83 ^y^	2681.45 ^z^	286.39	<0.001	<0.001	0.001

Note: 1 USD = 4.07 MYR currency conversion 5 March 2021, *MYR* Malaysian Ringgit. Estimations: income from live weight, RM 14.60/kg/animal; *Brachiaria* grass, RM 0.23/kg dry matter; Concentrate mixture, RM 1.11/kg; bypass fat, RM 3.82/kg. Diet A (control): 100% *Brachiaria decumbens*; Diet B: 70% *Brachiaria decumbens* + 30% concentrate); Diet C: 70% *Brachiaria decumbens* + 26% concentrate + 4% bypass fat; ^a,b,c,y,z^ means with different superscript letters in the same column are significantly different at *p <* 0.05. Abbreviation: SEM: standard error of means.

## Data Availability

Availability of data and equipment used and analysed during this study is available from the correspondence author on reasonable request.

## Abbreviations

CON: control; DMI: dry matter intake; FCR: feed conversion ratio; BCS: body condition score; ADG: average daily gain; GH: growth hormone; IGF-I: insulin-like growth factor-I; BW: body weight; BWG: body weight gain; h: hour; m: meter; MYR: Malaysian Ringgit; USD: United States Dollar; kg: kilogram; kcal: kilocalorie; ME: metabolize energy; LDL: low density lipoprotein; HDL: high density lipoprotein; g: gram; dl: decilitre; ng: nanogram; ml: millilitre; CP: crude protein; GHR: growth hormone receptor; IGFBP: insulin-like growth factor binding protein.
